# The sooner the better: clinical and neural correlates of impulsive choice in Tourette disorder

**DOI:** 10.1038/s41398-021-01691-2

**Published:** 2021-11-03

**Authors:** Cyril Atkinson-Clement, Astrid de Liege, Yanica Klein, Benoit Beranger, Romain Valabregue, Cecile Delorme, Emmanuel Roze, Emilio Fernandez-Egea, Andreas Hartmann, Trevor W. Robbins, Yulia Worbe

**Affiliations:** 1 Sorbonne Université, Institut du Cerveau - Paris Brain Institute - ICM, Inserm, CNRS, AP-HP, Hôpital de la Pitié Salpêtrière (DMU 6), Paris, France; 2 National Reference Center for Tourette syndrome, Assistance Publique des Hôpitaux de Paris, Groupe Hospitalier Pitié-Salpêtrière, F-75013 Paris, France; 3 Centre de NeuroImagerie de Recherche (CENIR), Sorbonne Université, UMRS975, CNRS UMR7225, ICM, F-75013 Paris, France; 4 Department of Neurology, Assistance Publique des Hôpitaux de Paris, Groupe Hospitalier Pitié-Salpêtrière, F-75013 Paris, France; 5 Cambridgeshire and Peterborough NHS Foundation Trust, Cambridge, UK; 6 Department of Psychiatry, University of Cambridge, Cambridge, UK; 7 Department of Psychology, University of Cambridge, Cambridge, UK; 8 Department of Neurophysiology, Saint Antoine Hospital, Assistance Publique des Hôpitaux de Paris, Paris, France

**Keywords:** Human behaviour, Psychiatric disorders, Neuroscience

## Abstract

Reward sensitivity has been suggested as one of the central pathophysiological mechanisms in Tourette disorder. However, the subjective valuation of a reward by introduction of delay has received little attention in Tourette disorder, even though it has been suggested as a trans-diagnostic feature of numerous neuropsychiatric disorders. We aimed to assess delay discounting in Tourette disorder and to identify its brain functional correlates. We evaluated delayed discounting and its brain functional correlates in a large group of 54 Tourette disorder patients and 31 healthy controls using a data-driven approach. We identified a subgroup of 29 patients with steeper reward discounting, characterised by a higher burden of impulse-control disorders and a higher level of general impulsivity compared to patients with normal behavioural performance or to controls. Reward discounting was underpinned by resting-state activity of a network comprising the orbito-frontal, cingulate, pre-supplementary motor area, temporal and insular cortices, as well as ventral striatum and hippocampus. Within this network, (i) lower connectivity of pre-supplementary motor area with ventral striatum predicted a higher impulsivity and a steeper reward discounting and (ii) a greater connectivity of pre-supplementary motor area with anterior insular cortex predicted steeper reward discounting and more severe tics. Overall, our results highlight the heterogeneity of the delayed reward processing in Tourette disorder, with steeper reward discounting being a marker of burden in impulsivity and impulse control disorders, and the pre-supplementary motor area being a hub region for the delay discounting, impulsivity and tic severity.

## Introduction

Tourette disorder (TD) is a childhood-onset neurodevelopmental disorder, characterised by multiple motor and vocal tics, which are often associated with several psychiatric comorbidities.

Enhanced sensitivity to reward [1], putatively resulting from hyperactivity of dopamine neurotransmission [2], was found in unmedicated patients with TD. It was suggested as one of the central mechanisms of TD pathophysiology, leading to dysfunction of basal ganglia-cerebellar-thalamo-cortical networks [3]. For instance, in reinforcement learning paradigms, TD patients outperformed the healthy controls (HC) [4] and learnt faster motor sequences associated with a higher reward [5]. Interestingly, the presence of co-morbid obsessive-compulsive disorders (OCD) impaired task learning similarly to antipsychotics [6].

Other facets of reward processing such as modification of the subjective value of a reward by introducing aspects of risk, effort, or delay [7, 8] have been addressed in only two studies. The first one was performed on a small sample of young persons with pure TD and no medication and showed a steeper delay discounting in TD patients in comparison to a control group only for rewards of large magnitude [9]. The second was carried out on both TD adolescents and adults using a computational approach and revealed temporal discounting only for adolescents’ patients in comparison to a matched control group which was not correlated to tics severity or co-morbidities [10]. Thus, it remains unclear whether abnormal reward processing in TD spreads beyond the reinforcement learning in adults with TD, if it is associated with specific clinical features and especially what are its functional brain correlates.

One of the factors influencing subjective reward evaluation is the introduction of a temporal delay to reward gratification. All animals, including humans, prefer a larger than a smaller reward and also prefer receiving it sooner than later [11]. Delayed discounting is a behavioural measure based on how rapidly a reward loses its value depending on its temporal distance or delay. A systematic preference for immediate reward or a steeper delayed hyperbolic discounting of reward is also referred to as an impulsive choice. Genetic genome-wide association studies have suggested a robust (up to 50%) heritability with increasing genetic influence on delayed discounting throughout brain development [12]. At a neural level, delayed discounting has been associated with the structure and function of the ventromedial prefrontal cortex, orbitofrontal cortex, temporal pole, and temporoparietal junction [13], insular cortex [14] and ventral striatum [15], all regions also associated with TD [16]. Delayed discounting has been suggested as a trans-diagnostic feature of numerous psychiatric disorders [17] and also recognised as a modifiable risk factor for behavioural and pharmacological clinical interventions [18].

The present study aimed to evaluate the delayed discounting of reward as a potential marker of abnormal reward processing in adults with TD, as well as its association with frequent psychiatric co-morbidities in this setting. In addition, this study is the first to explore the functional brain correlates of delay discounting in TD. We hypothesised that adult patients with TD would present a steeper delay discounting. We also addressed the question of brain functional correlates of delayed discounting in TD.

## Materials and methods

### Subjects

The present study was approved by the local ethics committee (CCP16163/C16-07) and preregistered on ClinicalTrial (https://clinicaltrials.gov/ct2/show/NCT02960698). We recruited 64 TD patients through the Tourette reference centre at the Pitié-Salpêtrière Hospital in Paris and 34 age- and gender-matched HC (2:1 matching). All participants gave their written consent to participate in the study. The exclusion criteria were lack of capacity or unwillingness to give consent for the study, evidence of either present or prior substance addiction (excluding nicotine; recreational cannabis use was allowed), a past or present history of psychosis, neurological symptoms other than tics for TD, childhood tics and Axis I psychiatric disorders for HC. The presence of co-morbidities was not used as an exclusion criteria since pure TD patients are rare and therefore less representative of the disease [19].

All participants were assessed for psychiatric disorders (Mini International Neuropsychiatric Interview, MINI [20]), impulse-control disorders (Minnesota Impulse Disorders Interview, MIDI [21]), general impulsivity (using the Barratt Impulsivity scale, BIS-11 [22]) and anxiety (State-Trait Anxiety Inventory, STAI [23]). Yale Global Tics Severity Scale (YGTSS [24]) was used to assess tics severity, and the presence or absence of psychiatric co-morbidities such as OCD and ADHD was evaluated using patient medical records and psychiatric evaluations prior to their inclusion in the study.

### Delay discounting questionnaire

To assess impulsive choices, we used a paper-and-pencil version of the Delay Discounting Questionnaire (DDQ) [25]. This test involves 27 items where the participants must choose between an immediate reward and a larger but delayed reward (from 11 to 85€ and from 7 to 186 days). Participants were instructed to answer each item of the DDQ without time constraint. Each item was associated with a specific *k* value which corresponds to the objective value of the delayed reward in comparison to the immediate reward (larger is the *k*, higher is the objective relevance of the delayed option). We identified the individual *k* value which corresponds to the geometrical mean of the maximal objective *k* value for immediate reward choice and the minimal objective *k* value of the delayed reward choice. The determination of this subjective *k* allows to identify the subjective delay discounting of a specific reward using the following hyperbola formula:

$${{{\mathrm{V}}}} = \frac{{{{\mathrm{A}}}}}{{1 + k{{{\mathrm{D}}}}}}$$

In this equation, A is the amount of the reward, *k* the subjective delay discounting value and D the delay to the reward.

### Neuroimaging data acquisition and pre-processing

Neuroimaging data were acquired on 3 T MRI scans (Siemens Prisma, Germany) with a 64-channel head coil. Two sequences were acquired: a T1-weighted image (MP2RAGE, TR = 5 s, TI = 700/2500 ms, fov = 256, 1 mm isotropic, Ipat acceleration of 3) and a resting-state functional MRI (rs-fMRI) of 11 min duration.

Rs-fMRI data were acquired with the eyes opened: the subjects were asked to fixate on a cross during the sequence acquisition, which was monitored by an eye tracker. Multi-echo echo-planar imaging (EPI) sequences were performed with a multi-slice, multi-echo acquisition scheme (TR = 1.9 s, TE = 17/36/56 ms, Ipat acceleration factor 2, Multi-band 2, isotropic voxel size 3 mm, dimensions = 66 × 66 in plane x 46 slices).

EPIs were processed with the MEICA toolbox (www.github.com/ME-ICA/me-ica/). This toolbox implements standard pre-processing steps: slice timing correction and realignment to the first volume driven by the first echo, co-registration to the anatomic volume. A single warp was applied to combine realignment and co-registration. Then, we performed a principal component analysis to reduce the dimensionality of the dataset by removing thermal noise, and an independent component analysis decomposition to separate BOLD (blood-oxygen-level-dependent) from non-BOLD components based on the echo time dependence of the BOLD component [26, 27]. After the independent component analysis, we obtained a dataset where thermal noise and physiological noise such as movements, breathing and cardiac artefacts were removed. Before analysis, to control for movements artefacts, we performed statistical analyses on movements metrics (reported in the Supplementary Fig. 1). Finally, we normalised the dataset using the flow field generated by the T1 processing from CAT12.

### Data analyses

Data were analysed using a data-driven approach with main objectives to identify (i) the subjects with impulsive choices in TD and (ii) identify brain functional correlates of the impulsive choices (see also Supplementary Fig. 2 for data analysis overview). Analyses were primarily focused on TD patients with impulsive choices since they represent the group of participants with abnormal behaviours and therefore with a possible abnormal brain processing of the delayed reward.

#### Behavioural data analysis

Behavioural analyses were performed with R [28]. Demographic and clinical groups comparisons were based on ANOVA and Chi-squared test when appropriate. Post-hoc analyses were corrected to multiple comparisons with the Tukey method. Cohen’s *d* effect size was also reported.

Subjective log(*k*) values of the DDQ (logarithm since non-normal distribution) were compared between group using ANOVA and Tukey post-hoc and correlated with clinical data. The 95% confidence interval of the HC group was used to identify two subgroups of TD patients, one with log(*k*) inside the 95% confidence interval of HC, which corresponded to similar to control group discounting in TD (TD-Sim) and one with log(*k*) higher than the 95% confidence interval which corresponded to the patients with impulsive choices (TD-Imp).

#### Resting-state functional connectivity analysis

Rs-fMRI neuroimaging analyses were performed using the Nistats, Nilearn and Sklearn modules implemented in Python [29] and SPM12 implemented in Matlab.

First, we performed independent component analyses (ICA) on the TD-Imp group, which generated twenty components. Second, for each of these components and all participants, we extracted the Hurst exponent, which represents the self-similarity of a time series [30–32]. Third, we computed the partial correlations between each of our Hurst exponent for the 20 components and the subjective log(*k*) to identify the possible relation between components networks and delay discounting in our TD-Imp patients. Last, we extracted signal from each regions of the components and performed connectivity analysis using partial correlation with the log(*k*) values to identify if one specific part of the component is involved in delay discounting. A flowchart for data analysis is provided in the Supplementary Fig. 2.

We also computed voxel-based morphometry on the Z-standardised fractional amplitude of low-frequency fluctuation (zfALFF, representing the spontaneous brain activity) for each of the different regions identified within the significant components. Threshold for significance was set at *p* < 0.05 following FWE correction at the peak voxel.

## Results

### Behavioural results

The final sample included 54 TD patients and 31 HC. Ten TD and three HC were excluded from the analysis due to missing data or bad quality of the MRI.

Direct comparison of the subjective log(*k*) values showed no significant differences between HC and all TD patients (F_(1;83)_ = 3.26; *p* = 0.074; *d* = 0.39), nor any effect of medication status (F_(2;82)_ = 2.24; *p* = 0.113; *d* = 0.47), ADHD (F_(2;82)_ = 2.74; *p* = 0.07; *d* = 0.52) or OCD comorbidities (F_(2;82)_ = 2.08; *p* = 0.131; *d* = 0.45). In all TD patients, we found no significant correlation with YGTSS/50 (t_(52)_ = 0.58; *p* = 0.566; *r* = 0.079; *d* = 0.16), the number of impulse-control disorder (ICD; t_(52)_ = 1.56; *p* = 0.124; *r* = 0.212; *d* = 0.43), BIS-11 total score (t_(52)_ = 0.98; *p* = 0.329; *r* = 0.135; *d* = 0.27), or STAI (t_(52)_ = 0.91; *p* = 0.368; *r* = 0.125; *d* = 0.25). However, we found a significant correlation with the motor subscale of the BIS-11 (t_(52)_ = 2.14; *p* = 0.037; *r* = 0.284; *d* = 0.59) while the attentional and non-planning subscales were not significant (t_(52)_ ≤ 0.33; *p* ≥ 0.74; *r* ≤ 0.045; *d* ≤ 0.091).

**Fig. 1:**
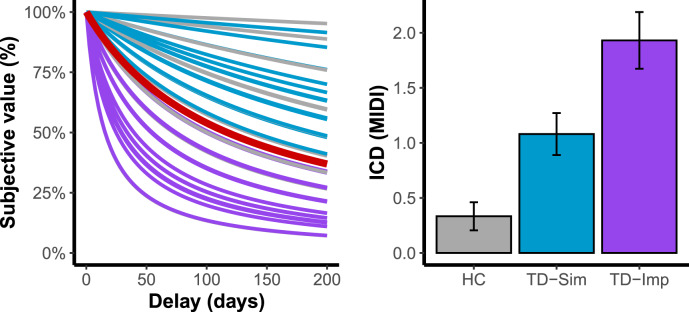
Representations of the TD subgroup classification. The left-hand panel represents the hyperbolic discounting curve for all participants, with the red curve corresponding to the 95% confidence interval for the healthy control group. Patients below this line were considered to make impulsive choices while patients above this line had performance comparable to that of HC. The right-hand panel displays the ICD score for each of the three subgroups. HC healthy controls, ICD impulse-control disorder, MIDI Minnesota impulse disorders interview, TD-Imp Tourette patients considered as having impulsive choices, TD-Sim Tourette patients considered as having not impulsive choices.

We then use a confidence interval for our hypothesis testing [33, 34]. After applying the 95% confidence interval in HC, we identify that the confidence interval of the mean was between −2.452 and −2.069. Therefore, we identified two groups of TD patients: the first with no differences from HC (*n* = 25; TD-Sim; subjective log(*k*) values ≤ −2.069) and the second group with a steeper delay discounting (*n* = 29; TD-Imp; subjective log(*k*) values > −2.069; Fig. 1).

As shown in Table 1, there were differences among the 3 groups in scores on BIS-11, STAI and MIDI scales, with the highest scores in the TS-Imp group. The two TD subgroups showed also a significant difference on MIDI scores (Fig. 1), where a TD-Imp subgroup showed the highest score. Specifically, TD-Imp group have a higher proportion of binge-eating disorder (F_(1;52)_ = 11.93; *p* = 0.001; *d* = 0.96) and intermittent explosive disorder (F_(1;52)_ = 4.76; *p* = 0.0336; *d* = 0.6). The two TD subgroups showed no difference in the severity of tics (YGTSS/50), percentage of patients with ADHD or OCD co-morbidities or percentage of patients under antipsychotic medication. In TD-Imp group, log(*k*) values significantly correlated with motor (t_(27)_ = 2.89; *p* = 0.007; *r* = 0.486; *d* = 1.11) and non-planning (t_(27)_ = 2.15; *p* = 0.041; *r* = 0.382; *d* = 0.83) subscales of the BIS-11 but not with the attentional subscale (t_(27)_ = −0.4; *p* = 0.69; *r* = −0.08; *d* = 0.15); (see also Supplementary Table 1 for detail of medication).

**Table 1:** Demographics and clinical characteristics of the participants.

	HC	TD-Sim	TD-Imp	*p* values
*N*	31	25	29	–
Gender (M/F)	22/9	20/5	23/6	0.66
Age	31.2 ± 10.5	30.6 ± 11.4	29.3 ± 9.9	0.77
BIS-11 (Total)	58.7 ± 9.7	65 ± 10.4	65.9 ± 10.8^a^	0.017
BIS-11 (attention)	15.8 ± 3.7	19 ± 4.1^a^	19.8 ± 4.9^a^	0.001
BIS-11 (motor)	20.3 ± 3.8	20.6 ± 3.1	21.6 ± 3.8	0.37
BIS-11 (non-planning)	22.5 ± 3.9	25.4 ± 5.5	24.4 ± 4.6	0.07
STAI	62.3 ± 14.6	78.6 ± 17.1^a^	82.8 ± 19.9^a^	<0.001
YGTSS/50	0	16.5 ± 7.4	16.1 ± 7.1	0.85
ICD (MIDI)	0.3 ± 0.7	1.1 ± 0.9^a,b^	1.9 ± 1.4^a,b^	<0.001
Medication (%)	0%	32%	38%	0.86
ADHD (%)	0%	40%	52%	0.56
OCD (%)	0%	28%	17%	0.53

*ADHD* attention-deficit hyperactivity disorder, *BIS-11* Barratt Impulsivity scale, *F* Female, *HC* healthy controls, *ICD* number of impulse-control disorders behaviours, *M* male, *MIDI* Minnesota impulse disorders interview, *OCD* obsessive-compulsive disorder, *STAI* state-trait anxiety inventory, *TD-Imp* patients with Tourette disorder and with impulsive choices, *TD-Sim* patients with Tourette disorder and without impulsive choices, *YGTSS/50* Yale global tics severity scale.

^a^Significantly different from HC after Tukey post-hoc.

^b^Significant differences between the two TD groups after Tukey post-hoc.

### Brain correlates of delay discounting

ICA performed on the TD-Imp group allowed identification of 20 distinct functional networks (reported in the Supplementary Fig. 3). After extracting time series activities, we computed the Hurst exponent for all components and all subjects. We found no significant differences among the 3 groups (*p* > 0.05).

In the TD-Imp group, we performed partial correlations between the 20 Hurst exponents of the metrics. We found no significant association with demographic and clinical data, but a significant partial correlation was found between the log(*k*) values and the Hurst exponents of the 7th independent component (t_(27)_ = −2.68; *p* = 0.027; slope = −0.688; *d* = 1.03).

Further decomposition analysis identified distinct regions in the 7th independent component as follows: the hippocampus and the angular gyrus bilaterally, the medial orbitofrontal gyrus, the right lateral orbitofrontal gyrus/anterior part of the insula, the right pre-supplementary motor area (pre-SMA), the middle and posterior part of the cingulate gyrus bilaterally, the right middle temporal gyrus, the left temporal pole, and the ventral striatum bilaterally (for details, see Supplementary Table 2). For this network, we performed functional connectivity analysis using a correlation matrix for all participants.

**Fig. 2:**
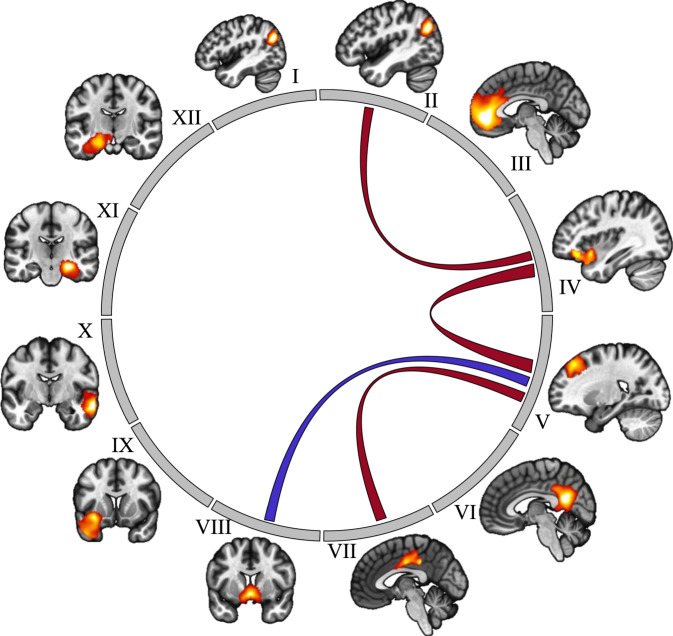
Representation of the 12 subparts of the 7th independent component and their relationships with the log(*k*) values of the TD subgroup with impulsive choices. Blue lines represent negative associations (higher the connectivity, lower the log(*k*) [i.e., lower the impulsive choices]) and red lines represent positive associations (higher the connectivity, higher the log(*k*) [i.e., higher the impulsive choices]). I: Angular gyrus left; II: Angular gyrus right; III: Medial orbitofrontal gyrus; IV: Lateral orbitofrontal gyrus/insula right; V: Pre supplementary motor area; VI: Posterior cingulate gyrus; VII: Middle cingulate cortex; VIII: Ventral striatum; IX: Temporal pole left; X: Middle temporal gyrus right; XI: Hippocampus right; XII: Hippocampus left.

For TD-Imp, partial correlations showed that log(*k*) values correlated positively with (Fig. 2): connectivity between the right pre-SMA and the right lateral orbitofrontal gyrus/anterior insula (t_(27)_ = 3.027; *p* = 0.008; slope = 0.603; *d* = 1.16) and with the posterior part of the middle cingulate gyrus bilaterally (t_(27)_ = 2.345; *p* = 0.032; slope = 0.506; *d* = 0.9) as well as with connectivity between the right orbitofrontal lateral gyrus/anterior insula and the left angular gyrus (t_(27)_ = 2.294; *p* = 0.036; slope = 0.497; *d* = 0.88); correlated negatively with connectivity between the right pre-SMA and the ventral striatum (t_(27)_ = 2.176; *p* = 0.045; slope = −0.478; *d* = 0.84).

**Fig. 3:**
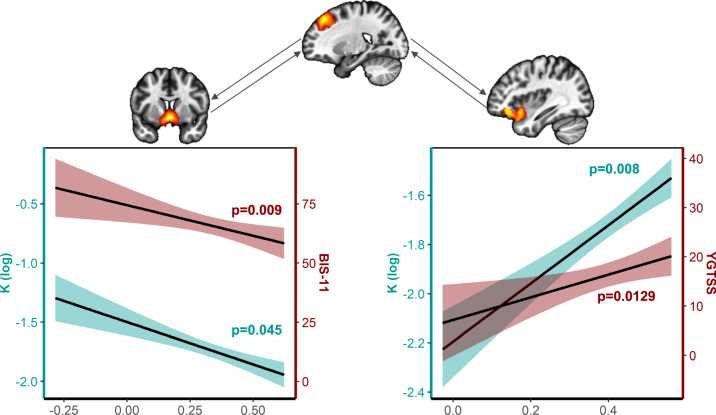
Correlations between the log(k), general impulsivity (BIS-11), tics severity (YGTSS/50) and brain functional connectivity. The left-hand panel represents the dual association between the log(*k*) (left ordinate axis) and the BIS-11 total score (right ordinate axis) with the connectivity between the ventral striatum and the right pre-SMA (i.e., higher the functional connectivity, lower the log(*k*) [i.e., impulsive choices] and lower the BIS-11 [i.e., general impulsivity]). The right-hand panel represents the dual association between the log(*k*) (left ordinate axis) and the YGTSS/50 (right ordinate axis) with the connectivity between the right orbitofrontal lateral gyrus/anterior insula and the right pre-SMA (i.e., higher the functional connectivity, higher the log(*k*) [i.e., impulsive choices] and higher the YGTSS/50 [i.e., tics severity]).

Regarding correlations with demographic and clinical data for the TD-Imp subgroup, we found that the connectivity between the right pre-SMA and the right orbitofrontal lateral gyrus/anterior insula was positively associated with tics severity, measured by the YGTSS/50 (t_(27)_ = 2.66; *p* = 0.0129; *r* = 0.456; *d* = 1.02; Fig. 3), and that the connectivity between the right pre-SMA and the ventral striatum was negatively associated with the BIS-11 scale (t_(27)_ = 2.79; *p* = 0.009; *r* = −0.473; *d* = 1.07; Fig. 3), especially with the motor (t_(27)_ = 3.53; *p* = 0.001; *r* = −0.562; *d* = 1.36) and the attentional (t_(27)_ = 2.07; *p* = 0.048; *r* = −0.369; *d* = 0.79) subscales. No differences between TD-Imp patients and both TD-Sim and HC groups were found in connections among the ROIs. However, we found a lower zfALFF values in right orbito-frontal cortex in TD-Imp group compared to controls (*x* = 6; *y* = 60; *z* = −16; *k* = 24; pFWE at the peak = 0.043; Supplementary Fig. 4).

## Discussion

Using a delayed discounting paradigm and fully data-driven neuroimaging analysis, we identified two groups in TD—those with similar performance in DDQ and those with steeper delay discounting. Clinically, the TD group with steeper delay discounting was characterised by a higher burden of impulse-control disorders (as measured by MIDI), a higher level of general impulsivity (as measured by the BIS-11 scale), but with no relationship to OCD, ADHD, medication or severity of tics.

At a neural level, steeper reward discounting in TD patients was underpinned by resting-state activity of a network composed of the orbito-frontal, cingulate, pre-SMA, temporal and insular cortices, as well as ventral striatum and hippocampus. Within this network, lower connectivity between pre-SMA and ventral striatum predicted higher impulsivity and steeper reward discounting and greater connectivity between pre-SMA and anterior insular cortex predicted steeper reward discounting and more severe tics.

This study had several limitations. Use of the pencil and paper questionnaire with hypothetical scenarios compared to a task with real rewards and delay could impact the results of this study. Indeed, we have not measured the choice time for each trial and so far, we were not able to evaluate the duration of the decisional process. Further study with a computerised discounting task could address this caveat. With regard to the real *vs*. hypothetical modalities, previous studies have shown little effect of the reward type on subjects’ choices, with a tendency to prefer a larger, delayed rewards in hypothetical scenarios compared to actual delays [35]. However, this limitation does not detract from the main conclusions of the study, namely heterogeneity of delayed reward processing in patients with TD with abnormal performance in a delayed discounting task being a marker of clinical impulsivity and impulse control disorders associated with TD rather than a neurocognitive marker of TD in general.

### Steeper delayed discounting characterised a sub-group of Tourette patients with a higher burden of impulsivity and impulse control disorders

Abnormal reward processing, in particular, greater reward sensitivity, has been suggested to be a behavioural marker of TD [1]. However, only two studies previously investigated delay discounting of reward in TD, using a task similar to that used in the present study, also showing steeper reward discounting, but only for large rewards and only for adolescents suffering from TD [9, 10]. In addition, as one of the previous study, we reported no significant effect of ADHD or OCD co-morbidities [10]. However, we found that connectivity between the right pre-SMA and the ventral striatum was related to both delay discounting and attentional impulsivity assessed with the BIS-11. Our present results are therefore not in favour of delay discounting as a neurocognitive marker of TD, suggesting instead that steeper delay discounting is a marker of ICD and general impulsivity burden frequently present in TD. Indeed, a positive relationship between ICD and delayed discounting has been previously shown in many other disorders such as gambling, substance use disorders and Parkinson’s disease [36–38]. We have recently shown that ‘waiting impulsivity’, a form of motor impulsivity occurring during action delay or restraint, correlated with tic severity in unmedicated patients with TD [39]. In conjunction with the present results, this highlights the motor vs. cognitive impulsivity dichotomy and heterogeneity in TD. On the one hand, the capacity to delay actions was related to tic severity. On the other hand, the capacity to choose delayed rewards was not connected to tics severity but is related more to burden in impulse control disorders, to general impulsivity and in some extent to the motor impulsivity assess through the BIS-11.

We were able to stratify patients with regard to co-morbidities and medication and showed no effect of antipsychotic medication or associated ADHD or OCD, the most frequent co-morbidities in TD. The absence of effect of frequent co-morbidities with TD was surprising, however a recent meta-analysis on children and adolescents with ADHD showed a similar pattern of decision-making across delay gratification and delay discounting tasks compared to typically developing individuals [40]. However, we found the correlation with the attentional subscale of the BIS-11 scale suggesting that some of the clinical aspects of ADHD could contribute to the performance in the TD-Imp group. Some studies have also pointed to the absence of differences in performance on these tasks in OCD [41]. Nonetheless, despite a large group of patients with TD included in this study, the overall number of patients with associated ADHD and OCD was rather small and further transdiagnostic studies are warranted to fully address the question of delay discounting across different neurodevelopmental disorders.

Finally, a meta-analysis of animal studies concerning effects of different dopaminergic drugs on delay discounting of reward showed that drugs with agonist effects at D1 and D2 receptors had no effect on discounting of reward, in contrast to D1 and D2 antagonists, which increased reward discounting [42]. As 28% of the patients and 80% of medicated patients included in this study were treated with aripiprazole, a partial dopaminergic receptor agonist, it was perhaps not surprising that we found no effect of this treatment on delay discounting.

### Neural network underpinning delay discounting of reward in TD

Several decisional mechanisms and related brain networks have been associated with delay discounting [43–46]. The reward evaluation network is usually associated with ventral striatal and prefrontal cortical activity, especially in the medial and ventro-medial regions, as well as the orbito-frontal and cingulate cortices. In this study we found a lower intrinsic activity in right orbito-frontal cortex in patients with steeper delay-discounting compared to controls. This is in line with animal studies, showing that lesions (and by consequence lower activity) of orbital cortex shifter the preferences for immediate reward [47].

The second cognitive control neural network is associated with activity of dorso-lateral and dorso-medial cortices, as well as parietal and cingulate cortex. The third, predictive and affective, network comprises temporal cortex, amygdala and hippocampus. All of these cortical and subcortical areas identified in our study were indeed related to delay discounting.

Specifically, connectivity of the pre-SMA with the posterior cingulate cortex and insular cortex, and anterior insular cortex with parietal cortex predicted a steeper discounting rate. Lower connectivity of pre-SMA with ventral striatum also predicted steeper discounting. Among these regions, in previous studies, the insular cortex exhibited activity during the choice decisional process [48] and during preference for immediate reward [49]. The value of the discounted stimulus at the time of choice was positively associated with the activity of the posterior cingulate cortex [50] and negatively associated with the activity of the inferior parietal cortex, which also tracks reward delay [51]. Ventral striatal activity was related to the evaluation of the future gain magnitude [51]. Finally, pre-SMA has not previously been associated with the decisional process per se [43], but is an important part of the motor response to the decisional process.

Overall, within the theoretical framework, the present results suggested that a propensity to steeper delay discounting in TD-Imp was likely a result of the interaction of brain regions implicated in cognitive control with reward evaluation network and motor response networks.

### Pre-SMA as a common region underpinning reward discounting, impulsivity and tics

Pre-SMA was a common region among the neural networks which functional connectivity was related to impulsivity, delay discounting and tic severity. Greater connectivity of the pre-SMA with ventral striatum correlated with a lower score of impulsivity and lower delay discounting. Pre-SMA is probably, the main one of several brain regions implicated in motor response inhibition [52]. Pre-SMA was also shown to be implicated in the representation of the action goals [53]. Modulation of pre-SMA activity resulted in the prevention of behavioural expression of impulsive actions without effect on their generation [54]. Consequently, greater connectivity of pre-SMA with the ventral striatum could result in superior motor control over the reward system, as higher activity of the ventral striatum predicted impulsive choices in HC [55].

In contrast, greater connectivity of pre-SMA with insular cortex correlated with steeper delay discounting and more severe tics. The insular cortex has been related to both generation of the premonitory urges and tics in TD [56, 57], and greater insular connectivity with several cortical areas, including the SMA complex, predicted more severe tics [58]. As discussed previously, insular cortex activity also underpinned choice in delay discounting and preference for immediate choice, likely through its interaction with a motor system in general and pre-SMA in particular.

## Conclusion

In conclusion, steeper delay discounting is a marker of ICD burden in TD rather than a cognitive endophenotype of the disorder as was suggested in some previous studies. At a neural level, the steeper reward discounting was related to a network composed of the orbito-frontal, cingulate, pre-SMA, temporal and insular cortices, as well as ventral striatum and hippocampus. These results are in line with the brain networks known to be involved in reward processing through a preference for immediate reward, as well as with the cognitive control and affective neural networks. In particular, the pre-SMA play a node role in TD, underpinning reward discounting, impulsivity and tics.

## Supplementary information

Supplementary materials

## Data Availability

The conditions of our ethics approval do not permit public archiving of individual anonymised raw data. Readers seeking access to the data should contact the lead author Dr. Worbe. Access will be granted to named individuals in accordance with ethical procedures governing the reuse of sensitive data. Specifically, requestors must obtain a specific authorisation from the ethics committee. Regarding scripts for experimental task and statistical analyses, they are available upon request from the corresponding author.
