# Supplementary material for: The sooner the better: clinical and neural correlates of impulsive choice in Tourette disorder
Source: Transl Psychiatry. 2021 Nov 3;11:560. doi: 10.1038/s41398-021-01691-2 (PMC8566507; doi:10.1038/s41398-021-01691-2)
Supplement: Supplementary file 1 — Supplementary materials [file 41398_2021_1691_MOESM1_ESM.docx]

**Supplementary Table 1. Medication of patients with Tourette disorder.**

| **Third generation neuroleptics** | **Second generation neuroleptics** | **Typical neuroleptics** | **Others** |
| --- | --- | --- | --- |
| **TD-Sim group (n=8/25)** | | | |
| Aripiprazole (2.5mg/d) |  |  |  |
| Aripiprazole (5mg/d) |  |  |  |
| Aripiprazole (5mg/d) |  |  |  |
| Aripiprazole (10mg/d) |  |  |  |
| Aripiprazole (5mg/d) |  |  | Fluoxetine (20mg/d) |
| Aripiprazole (15mg/d) |  |  | Mianserin (60mg/d)  Fluoxetine (40mg/d)  Prazepam (10mg/d) |
|  |  | Haloperidol (0.5mg/d) | Escitalopram (10mg/d) |
|  |  | Pimozide (0.25mg/d) |  |
| **TD-Imp group (n=11/29)** | | | |
| Aripiprazole (1.25mg/d) |  |  |  |
| Aripiprazole (2.5mg/d) |  |  |  |
| Aripiprazole (5mg/d) |  |  |  |
| Aripiprazole (5mg/d) |  |  |  |
| Aripiprazole (5mg/d) |  |  |  |
| Aripiprazole (5mg/d) |  |  |  |
| Aripiprazole (5mg/d) |  |  |  |
| Aripiprazole (5mg/d) |  |  |  |
| Aripiprazole (10mg/d) |  |  |  |
|  | Risperidone (1mg/d) |  |  |
|  | Risperidone (2mg/d) |  | Topiramate (100mg/d) |

**Supplementary Table 2. Twelve distinct territories of the 7th independent component related to decisional impulsivity in the TD-Imp group.**

| **Anatomical regions** | **Laterality** | **Cluster size** | **Peak MNI coordinates** | | |
| --- | --- | --- | --- | --- | --- |
|  |  |  | **X** | **Y** | **Z** |
| Ventral striatum | R/L | 2066 | 0 | 12 | -8 |
| Middle cingulate cortex | R/L | 716 | -2 | -34 | 36 |
| Posterior cingulate cortex | R/L | 3082 | -6 | -54 | 18 |
| Medial orbitofrontal gyrus | R/L | 7226 | 0 | 52 | -6 |
| Lateral orbitofrontal gyrus / Insula | R | 849 | 34 | 34 | -12 |
| Pre supplementary motor area | R | 917 | 18 | 34 | 44 |
| Hippocampus | R | 2811 | 26 | -20 | -16 |
| Hippocampus | L | 3593 | -24 | -22 | -16 |
| Angular gyrus | R | 449 | 48 | -62 | 30 |
| Angular gyrus | L | 566 | -42 | -72 | 34 |
| Middle temporal gyrus | R | 2188 | 60 | -10 | -16 |
| Temporal pole | L | 3083 | -58 | -10 | -14 |

*L: Left; R: Right.*


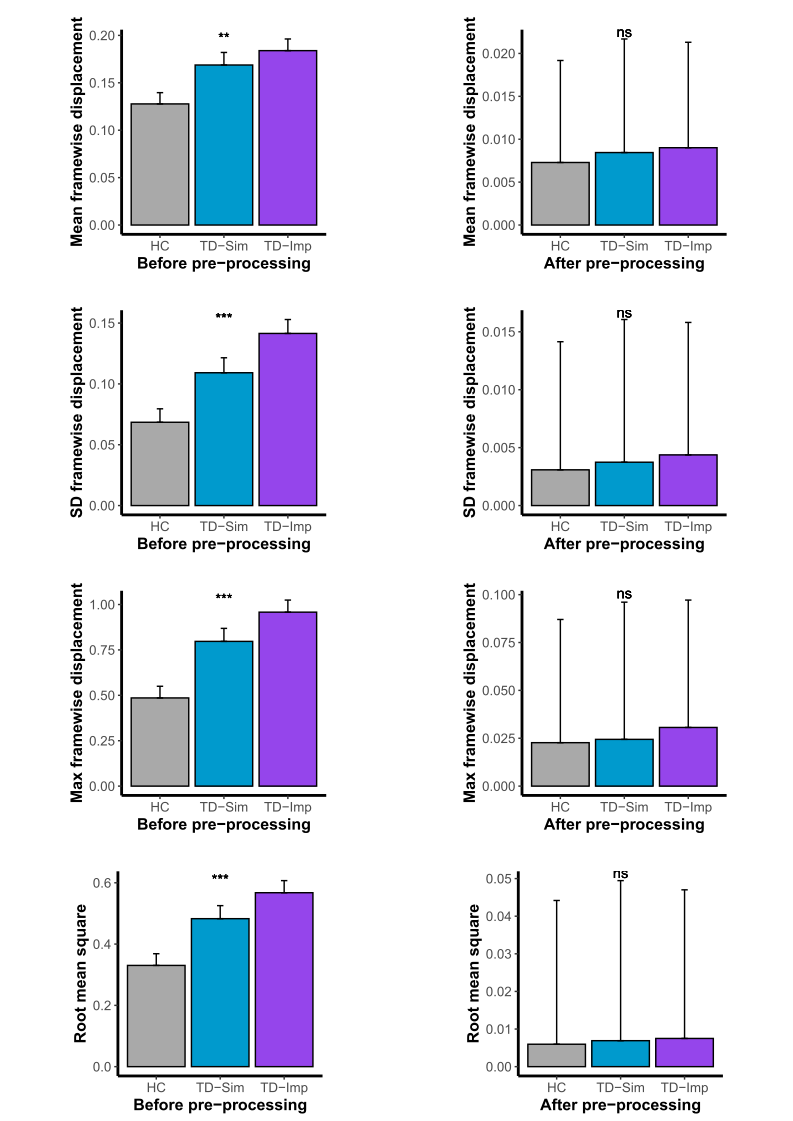


**Supplementary.Fig.1. Movements’ artefacts in each group and for each of 4 metrics before (left-hand panel) and after resting state images pre-processing (right-hand panel) No groups differences were found after pre-processing.**

*HC: Healthy controls; SD: Standard Deviation; TD-Imp: Tourette patients considered as having impulsive choices; TD-Sim: Tourette patients considered as having not impulsive choices.*


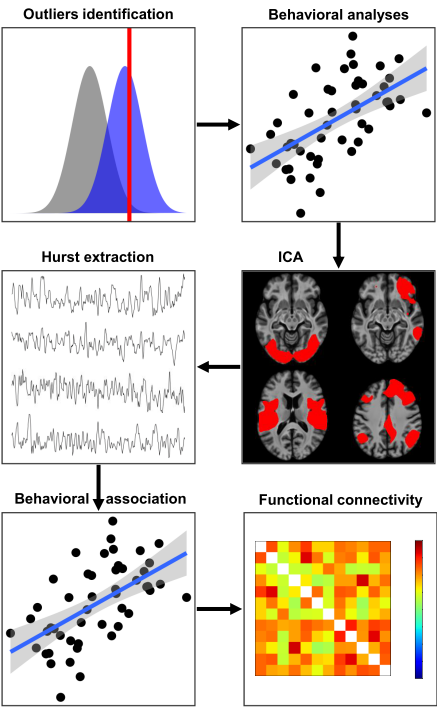


**Supplementary.Fig.2. Flowchart of the data analyses. First, we used 95% confidence interval to identify TD patients with impulsive choices (TD-Imp) and TD patients similar (TD-Sim) compared to performance of control group. On the second stage, we performed behavioral analyses to identify clinical differences between the three groups (i.e., HC, TD-Sim, TD-Imp). Then, we identified 20 functional brain networks using ICA in TD-imp group and we extracted Hurst exponent for the 20 ICA and for all participants. We performed behavioral analyses to identify if one or several functional brain networks were related to impulsive choices in the TD-Imp subgroup and if they were several differences with the two other groups. Last, we performed functional connectivity between within the subcomponents of the ICA which were associated to impulsive choices in the TD-Imp subgroup in order to identify a specific network which could explain impulsive choices in this group.**


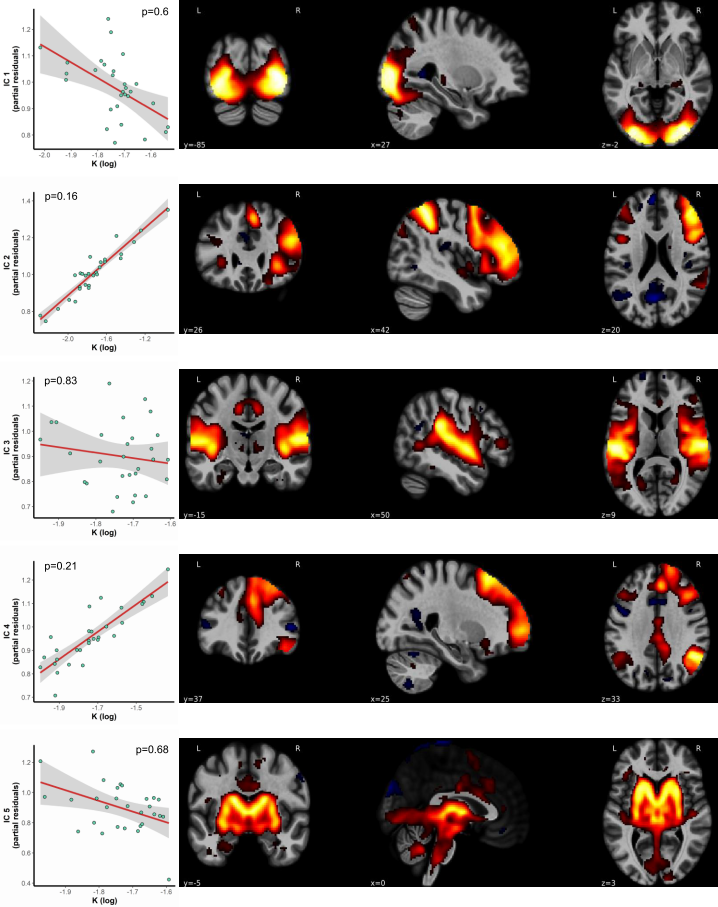


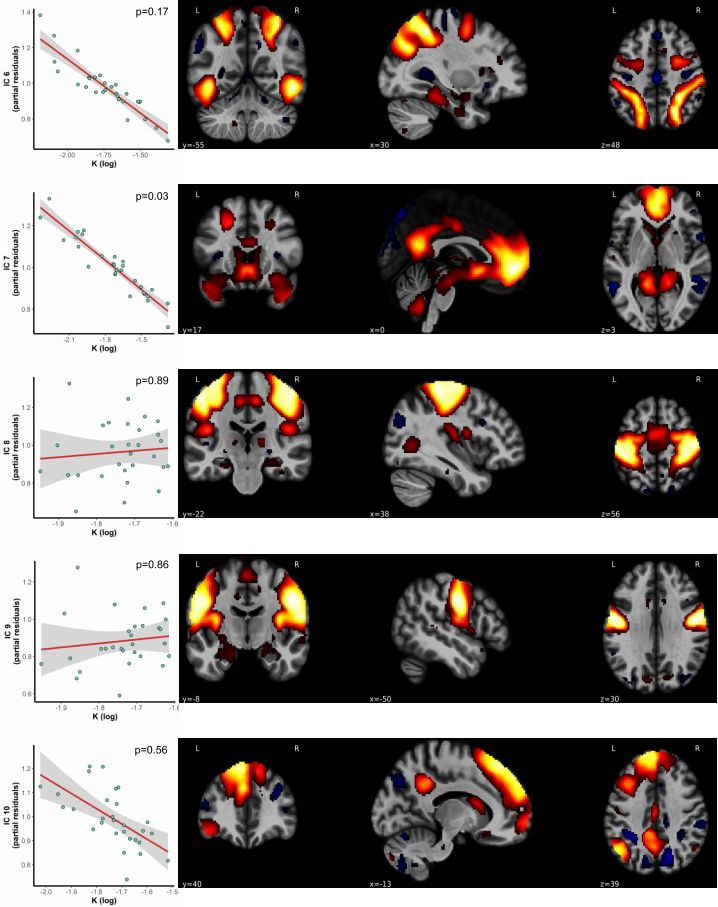


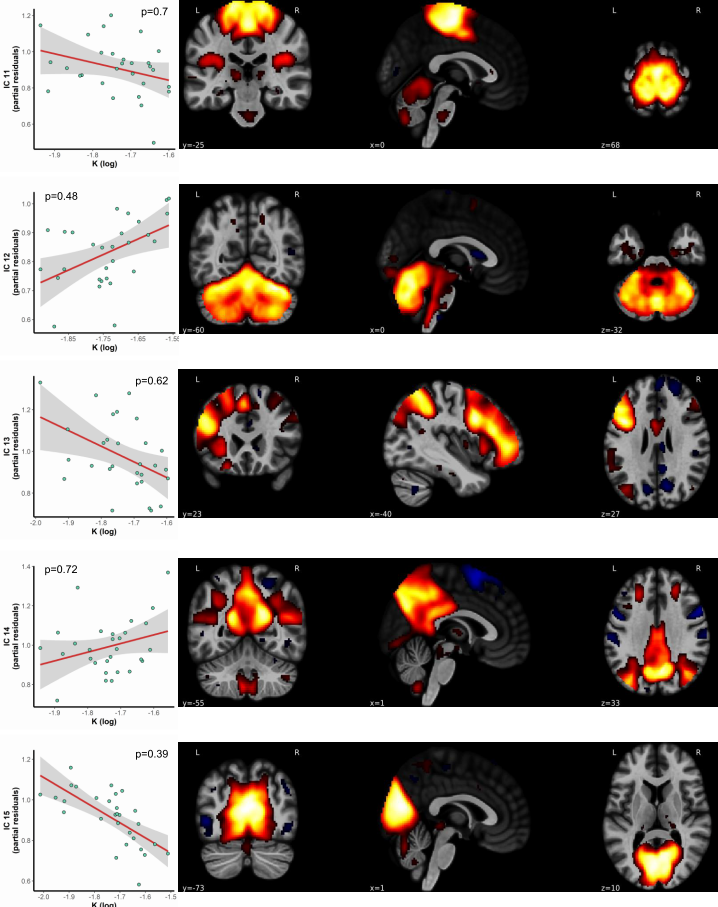


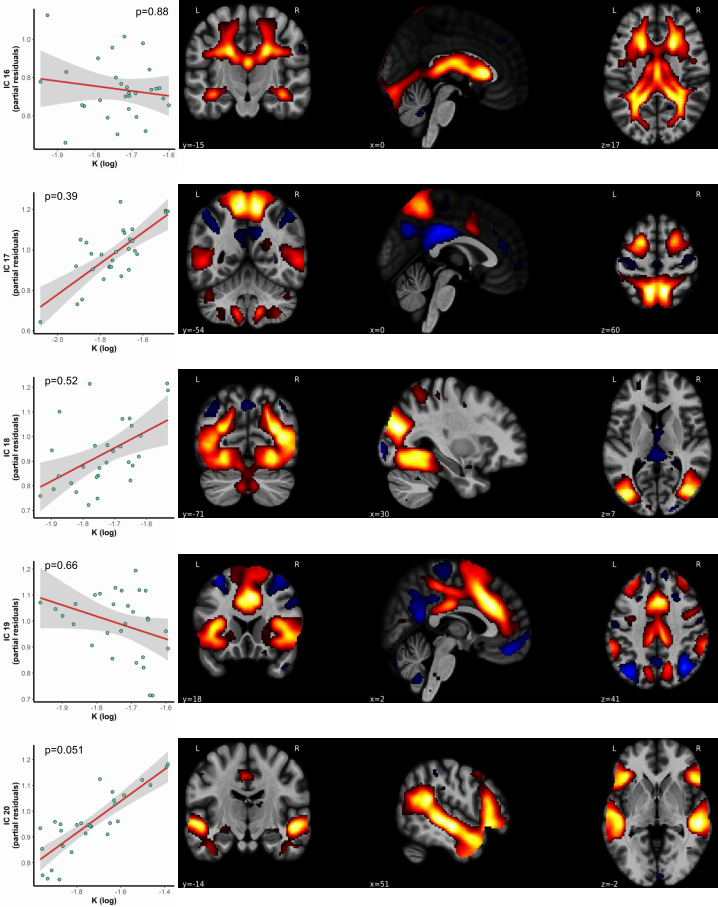


**Supplementary.Fig.3. 20 distinct functional networks identified by the ICA on the TD-Imp group and partial correlations between the Hurst exponent and the log(k).**


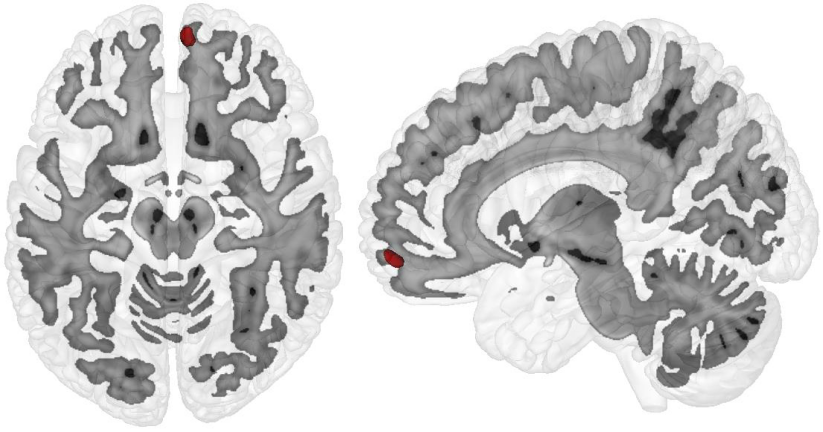


**Supplementary.Fig.4. Decreased zfALFF in the right orbito-frontal cortex for the TD-imp group in comparison to the control group.**
